# A commentary on ‘Distribution of coronal plane alignment of the knee classification in Chinese osteoarthritic and healthy population: a retrospective cross-sectional observational study’

**DOI:** 10.1097/JS9.0000000000001433

**Published:** 2024-04-09

**Authors:** Ao Xiong, Fei Luo, Hong Lu

**Affiliations:** a Department of Radiology, Central Hospital Affiliated to Chongqing University of Technology; b Department of Orthopaedics, First Affiliated Hospital, Army Medical University (Third Military Medical University), Chongqing, People’s Republic of China

*Dear Editor*,

It was with great interest that we read the article published in the International Journal of Surgery. Gao *et al*.^1^ conducted a retrospective cross-sectional observational study on patients with osteoarthritis (OA). According to research, types I and II were the most common distributions in Chinese populations, and OA progression may result in alterations to the Coronal Plane Alignment of the Knee (CPAK) categorization.

OA is a disease that affects the joint and related tissues^2^. It gradually deteriorates the articular cartilage, subchondral bone, and surrounding synovial structures. OA affects ~250 million individuals worldwide, or 4% of the total population, and is one of the 50 most prevalent aftereffects of illnesses and injuries. Eighty-three percent of the cases of OA worldwide are caused by knee OA. The recommended course of treatment for end-stage OA is total knee arthroplasty (TKA)^3^. According to a thorough analysis of Medicare patients, the annual TKA utilization rate varied from 287 006 in 2006 to 301 956 in 2010. It is anticipated that the need for TKA would skyrocket in the ensuing decades.

TKA is one of the most common orthopedic procedures done globally; among its objectives is a joint that is pain-free and functional as well as high patient satisfaction^4^. The three femoral kinematic axes are restored during TKA, together with the restoration of the native joint line and kinematics. These alignment procedures are mechanical alignment (MA), kinematic alignment (KA), inverse kinematic alignment (iKA), and restricted kinematic alignment (rKA). The ideal coronal alignment aim for TKA patients has long been a topic of discussion. Neutral coronal alignment and horizontal joint line obliquity (JLO) are the goals of MA in order to distribute loads evenly and have a high long-term survival rate. However, it frequently leads to inadvertent changes in the constitutional alignment of patients, and surgical resection precision mistakes can happen. In long-term follow-up, KA has demonstrated improved soft-tissue balance and great long-term surgical outcomes with the goal of restoring native (prearthritic) alignment. Determining a person’s prearthritic alignment and establishing a reasonable alignment goal are still unclear, though.

While it is not always necessary for the first-line diagnosis, conventional radiography is frequently used for the instrumental diagnosis of OA. This type of imaging can serve as a useful adjunct to clinical tests. Kellgren and Lawrence (KL) published a description of the initial systematic attempts to create a radiographic categorization scheme for OA in 1957. According to the study, among the diarthrodial joints they looked at, the tibiofemoral joint of the knee had the highest intraobserver correlation coefficient (r=0.83; range of all joints studied, 0.42–0.88) and the highest interobserver correlation coefficient (r=0.83; range of all joints studied, 0.10–0.83)^5^. By then, a simple method to ascertain the prearthritic alignment is to classify knee type in the coronal plane. MacDessi *et al*. recently presented the CPAK classification to the literature. This classification uses the arithmetic hip-knee-ankle angle (aHKA) and JLO to split lower extremity alignment into nine groups. Lower extremity alignment has been found to differ among populations as a result of CPAK research carried out in several nations. Based on constitutional coronal alignment and JLO, the CPAK categorization divides knee alignment into nine phenotypes. Neutral coronal alignment and horizontal JLO are the outcomes of using CPAK Type V as the alignment target for MA, regardless of the patient’s constitutional knee phenotype^4^.

The distribution of the CPAK classification in Chinese populations with OA and those in good health is the aim of this study. This novel approach provides insightful guidance and inspiration for the ongoing development of this field of study. But there were certain limitations. Despite recruiting a sufficient number of participants, the study’s single-center design limits how broadly the findings may be applied to the whole population. Results from research including several centers from various parts of the nation can be obtained that more accurately represent the population of China. Furthermore, it is possible to track alterations in lower limb alignment over time by doing long-term follow-ups of healthy individuals. To distinguish between ethnic and sexual variations in CPAK types, more research is required. An enhanced comprehension of the variety in knee phenotypes at the population level could potentially empower orthopaedic surgeons to provide a more customized knee arthroplasty procedure.

## Ethical approval

Not applicable.

## Consent

Not applicable.

## Author contribution

A.X.: study concept or design, data collection, data analysis or interpretation, and writing the paper; F.L.: data collection; H.L.: study concept or design, writing and revise the paper.

## Conflicts of interest disclosure

This manuscript is a comment without conflicts of interest.

## Research registration unique identifying number (UIN)

Not applicable.

## Guarantor

Hong Lu.

## Data availability statement

This manuscript is a comment. Do not need data availability statement. But all the data during the current study are publicly available.

## Provenance and peer review

This manuscript is a comment without being invited.
